# Insulinotropic and Muscle Protein Synthetic Effects of Branched-Chain Amino Acids: Potential Therapy for Type 2 Diabetes and Sarcopenia

**DOI:** 10.3390/nu4111664

**Published:** 2012-11-08

**Authors:** Ralph J. Manders, Jonathan P. Little, Scott C. Forbes, Darren G. Candow

**Affiliations:** 1 Exercise Physiology Research Group, Department of Kinesiology, KU Leuven, Heverlee, B-3001, Belgium; Email: ralph.manders@faber.kuleuven.be; 2 School of Health and Exercise Sciences, University of British Columbia Okanagan, Kelowna, British Columbia, V1V 1V7, Canada; Email: jonathan.little@ubc.ca; 3 Faculty of Physical Education & Recreation, University of Alberta, Edmonton, Alberta, T6G 2R3, Canada; Email: scforbes@ualberta.ca; 4 Faculty of Kinesiology and Health Studies, University of Regina, Regina, Saskatchewan, S4S 0A2, Canada

**Keywords:** amino acids, leucine, skeletal muscle, protein synthesis, insulin secretion

## Abstract

The loss of muscle mass and strength with aging (*i.e.*, sarcopenia) has a negative effect on functional independence and overall quality of life. One main contributing factor to sarcopenia is the reduced ability to increase skeletal muscle protein synthesis in response to habitual feeding, possibly due to a reduction in postprandial insulin release and an increase in insulin resistance. Branched-chain amino acids (BCAA), primarily leucine, increases the activation of pathways involved in muscle protein synthesis through insulin-dependent and independent mechanisms, which may help counteract the “anabolic resistance” to feeding in older adults. Leucine exhibits strong insulinotropic characteristics, which may increase amino acid availability for muscle protein synthesis, reduce muscle protein breakdown, and enhance glucose disposal to help maintain blood glucose homeostasis.

## 1. Introduction

The progressive loss of muscle mass and strength with aging, often referred to as sarcopenia [1] not only decreases overall health, but also increases the dependency on others during activities of daily life and, as such, reduces overall quality of life. Sarcopenia is a multifactorial process characterized by changes in muscle fiber morphology, muscle contractile and protein kinetics, and insulin sensitivity (for reviews see [2,3,4,5]). One main contributing factor towards the age-related loss in muscle mass and strength is the reduced ability to increase skeletal muscle protein synthesis in response to feeding, referred to as “anabolic resistance” (for reviews see [4,6]). Interestingly, ingestion of branched-chain amino acids (BCAA), primarily leucine, increases the activation of the mammalian target of rapamycin (mTOR) signaling pathways involved in muscle protein synthesis via insulin-dependent and independent pathways [7]. High doses of leucine may therefore help overcome “anabolic resistance” to feeding and have a favorable effect on muscle protein synthesis and subsequent maintenance of muscle mass with aging. The rates of muscle protein synthesis are relatively maintained in aging adults [8,9,10]. Leucine exhibits strong insulinotropic characteristics [11,12], which can increase amino acid availability for muscle protein synthesis, inhibit muscle protein breakdown resulting in greater net muscle protein balance over time and also enhance glucose disposal to help maintain blood glucose homeostasis. The purpose of this review is to highlight the potential beneficial health effects of BCAA, primarily leucine, on aging muscle metabolism. We will particularly highlight the role of BCAA in insulin resistance and type 2 diabetes, conditions of which sarcopenia may be a major contributing risk factor. 

## 2. Branched Chain Amino Acids

The branched chain amino acids (leucine, isoleucine, and valine) account for 14%–18% of the total amino acids in skeletal muscle protein [13,14]. It is well known that amino acids, including the BCAA, are required for maintenance of muscle health in older adults [15]. At rest, BCAA, and particularly leucine, have an anabolic effect through enhanced protein synthesis and/or a decreased rate of protein degradation [16,17,18,19], resulting in a positive net muscle protein balance. Infusion of BCAA in humans elevated the phosphorylation and activation of p70S6 kinase and 4E-BP1 in skeletal muscle [20,21]. Both p70S6 kinase and 4E-BP1 are downstream components of the mTOR signaling pathway, which controls RNA translation and protein synthesis, and is recognized as a central node in support of muscle hypertrophy [22,23,24]. Leucine ingestion is involved in the direct phosphorylation and activation of mTOR in skeletal muscle [25,26], further enhancing the protein synthetic response. However, changes in the rates of muscle protein synthesis are relatively transient unless sufficient amounts of essential amino acids are provided [27], either through normal dietary patterns or supplementation. When BCAA were consumed during and following an acute bout of knee extensor resistance exercise, Karlsson and colleagues [28] found an enhanced (3.5-fold) elevation in p70S6K phosphorylation during recovery in young healthy men compared to resistance exercise alone. The acute exercise-induced p70S6k activity has been shown to correlate with skeletal muscle hypertrophy following 6 weeks of resistance training [29]. In addition, it has been shown that BCAA can attenuate muscle wasting through interaction with the ubiquitin proteasome pathway [30]. This response may partially involve the protein kinase Akt/PKB pathway, which is known to phosphorylate the transcription factor forkhead box class-O (FoxO), that signals downstream to two major ubiquitin ligases atrogin-1 and muscle RING-finger protein (MuRF-1) involved in muscle atrophy [31,32,33]. 

Aging is known to suppress muscle protein synthesis, especially the synthetic response after feeding, which may alter net muscle protein balance leading to sarcopenia. Although sarcopenia is a multi-factorial affliction [3], amino acids and especially leucine, could play a major role in attenuating the age-related loss in muscle mass and strength. Splanchnic sequestration of leucine following feeding is 50% higher in older *vs.* younger adults and the rates of muscle protein synthesis are decreased with aging [34], termed “anabolic resistance” [35,36]. Therefore, older adults may require additional dietary protein with greater leucine concentration to counteract muscle wasting over time. Supplemental leucine ingestion has been shown to overcome resistance to the anabolic effects of amino acid consumption [37], providing evidence that leucine supplementation may be beneficial for preserving muscle mass with aging [34,37,38]. For example, the combination of leucine (2.5 g) and casein protein (20 g) elevated the rates of muscle protein synthesis for up to 6 h in older men compared to casein protein ingestion alone [39]. The co-ingestion of leucine (10 g/L) and whey protein (60 g/L) following an acute bout of lower body resistance exercise (6 sets of 10 repetition for leg press and leg extension) in eight older men (75 ± 1 years) significantly increased the rates of muscle protein synthesis and whole-body protein balance [40]. Furthermore, Casperson *et al.* [41] showed that 2 weeks of leucine supplementation (12 g/day) elevated the muscle protein synthetic response (*i.e.*, augmented mTOR/p70S6K signaling) compared to a standardized meal in older adults without having any effect on lean tissue accretion. It is important to note that acute studies examining phosphorylation or insulin availability after resistance exercise and/or amino acid ingestion are primarily used to predict longer-term training outcomes (*i.e.*, skeletal muscle hypertrophy) and as such, there may be a disconnect between these anabolic signals and end-point measures of protein synthesis [42]. Nevertheless, insulinotropic effects of leucine and/or BCAA may help to improve net muscle protein balance by increasing muscle protein synthesis [31,39,40], decreasing muscle protein breakdown [42], or both. This may be particularly important in long-standing T2D patients where insulin levels are chronically low. Further work, particularly longer-term studies, are warranted to determine if BCAA or leucine have the potential to reverse or prevent sarcopenia, enhance muscle function, and raise the overall quality of life for aging adults.

## 3. Insulinotropic Properties of Amino Acids

Next to the anabolic properties of BCAA on muscle health, amino acids can also have profound effects on insulin production/secretion, which could further augment the anabolic response and also be used as a modulator of glucose homeostasis. 

**Figure 1:**
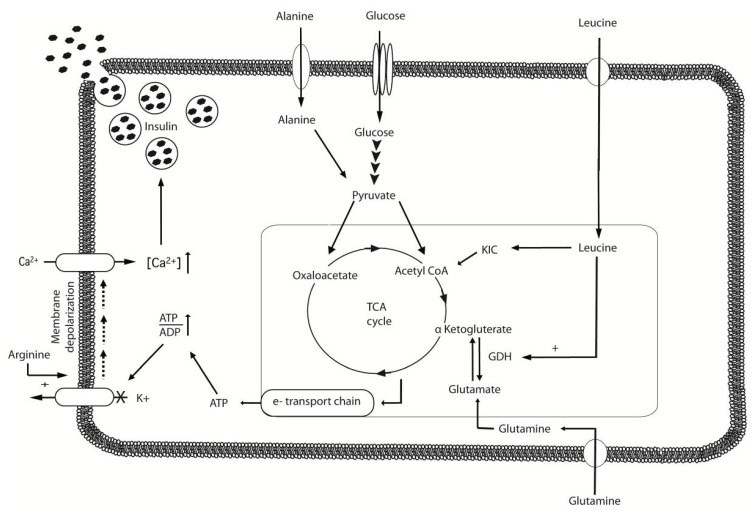
Simplified overview of amino acid induced insulin secretion in the β-cell. Glucose is metabolized in the cell via glycolysis into pyruvate, which is subsequently metabolized further by the tricaboxylic acid cycle (TCA cycle) to form ATP. Increased ratios of ATP/ADP result in depolarization of the plasma membrane through closure of ATP-sensitive K^+^ channels. This depolarization opens voltage activated Ca^2+^ channels, leading to increased concentrations of intracellular Ca^2+^ ([Ca^2+^]_i_) and subsequent insulin exocytosis. Intracellular catabolism of all metabolizable amino acids will increase the intracellular ATP/ADP ratio, thereby closing ATP-sensitive K^+^ channels, leading to the depolarization of the plasma membrane. Leucine both induces and enhances pancreatic β-cell insulin secretion through oxidative decarboxylation and allosteric activation of glutamate dehydrogenase (GDH) increasing ATP/ADP. Leucine can also be transaminated to α-ketoisocaproate (KIC) that is converted into acetyl-CoA before entering the TCA-cycle. Amino acids such as arginine can directly depolarize the plasma membrane of the β-cell, opening up voltage activated Ca^2+^ channels leading to insulin secretion. Adapted from Newsholme *et al.* [86].

The insulinotropic properties of amino acids or protein were reported for the first time in the 1960s [43,44], and have since been confirmed in healthy subjects [45] and type 2 diabetes patients [46,47,48]. In a series of studies, Floyd and co-workers [49,50,51,52,53] reported strong insulinotropic responses following the intravenous administration of various free amino acids. A strong synergistic stimulating effect on insulin release was observed when leucine and arginine were infused in combination with glucose [11]. Furthermore, numerous *in vitro* studies using primary pancreatic islet cells or β-cell lines have reported strong insulinotropic effects for (among others) leucine, isoleucine, arginine, alanine and phenylalanine [52,54,55,56,57,58,59,60,61]. The mechanisms by which these amino acids stimulate insulin secretion tend to be diverse and have not yet been fully elucidated [62]. Figure 1 provides a simplified overview of amino acid induced insulin secretion. In the presence of glucose, amino acids such as arginine have been shown to stimulate insulin secretion by directly depolarizing the plasma membrane of the β-cell [54], which opens up voltage activated Ca^2+^ channels, leading to the influx of Ca^2+^ and subsequent insulin exocytosis [57,62]. Other amino acids may modulate their insulinotropic properties through activating Ca^2+^ channels by their co-transport with Na^+^ [57,63]. Furthermore, intracellular catabolism of all metabolizable amino acids will increase the intracellular ATP/ADP ratio, thereby closing ATP-sensitive K^+^ channels, which can also lead to the depolarization of the plasma membrane [62,64,65]. Both *in vivo* and *in vitro* work has identified leucine as a particular interesting insulin secretagogue as it both induces and enhances pancreatic β-cell insulin secretion through its oxidative decarboxylation, as well as by its ability to allosterically activate glutamate dehydrogenase [60,62,66,67] which increases ATP/ADP ratios by increasing TCA-cycle fluxes resulting in depolarization of the plasma membrane through closure of ATP-sensitive K^+^ channels. Furthermore, leucine can be transaminated to α-ketoisocaproate which in turn is converted into acetyl-CoA before entering the TCA-cycle [68]. These findings are in line with recent *in vivo* observations, showing co-ingestion of relatively small amounts of free leucine to further augment the insulin response following the combined ingestion of carbohydrate and protein in healthy men [69]. Xu *et al.* [60] reported that the same signals that stimulate insulin release are also likely to be responsible for the leucine-induced activation of the mammalian target of rapamycin (mTOR) signaling pathway in the pancreatic β-cell. The potency of leucine to activate protein synthesis by interacting with the mTOR signaling pathway has been proposed to enhance β-cell function through the maintenance of β-cell mass. As such, the insulinotropic properties of amino acids (and leucine in particular) can therefore be of great clinical relevance in the treatment of type 2 diabetes or any state where there is a certain level of insulin resistance (e.g., aging) or hyperglycemia. Increasing endogenous insulin secretion with amino acids could therefore accelerate blood glucose disposal resulting in a better glycemic control. In longstanding type 2 diabetes patients, hyperglycemia is no longer accompanied by a compensatory hyperinsulinemia and as such, it is generally assumed that the capacity of the β-cell to secrete insulin is severely impaired due to several defects [70]. These defects include a reduced early insulin secretory response to oral glucose, a reduced ability of the β-cell to compensate for the degree of insulin resistance, a decline in the glucose-sensing ability of the β-cell, and a shift to the right in the dose-response curve relating glucose and insulin secretion, which are all indicative of a progressive insensitivity of the β-cell to glucose [71]. All these defects involve glucose-sensing and -signaling pathways in the β-cell. Although insulin secretion in response to carbohydrate intake is impaired in type 2 diabetes patients, it has been shown that the combined ingestion of a protein/amino acid mixture with carbohydrates can increase the plasma insulin response up to 4-fold [72,73]. This indicates that although the sensitivity of the pancreas to carbohydrate intake is significantly reduced in type 2 diabetes patients, the capacity to secrete insulin in response to stimuli like amino acids is still intact. Therefore, it can be concluded that the defects in the insulin response after a meal or glucose load in these patients can mainly be attributed to the reduced sensitivity of the β-cell to glucose, and not an overall defect in the capacity to produce and/or secrete insulin. For this reason it can also be assumed that the potential of amino acids is the greatest in longstanding type 2 diabetes patients as they are no longer in a state of hyperinsulinemia, in contrast to recently diagnosed patients where hyperglycemia is accompanied with hyperinsulinemia. Before amino acid supplementation or modulation can be considered an effective nutritional intervention in the treatment of type 2 diabetes, the mere increase in endogenous insulin production alone is not sufficient. In order to improve glycemic control (*i.e.*, lower blood glucose concentrations), the increased endogenous insulin secretory response should be able to overcome the level of insulin resistance and effectively lower plasma glucose concentrations by increasing the glucose disposal rate from the circulation. Using stable isotope glucose tracers, it has been shown that even though plasma glucose disposal was severely impaired in the type 2 diabetes patients, the addition of an amino acid/protein mixture increased plasma glucose disposal significantly and results in a lower glycemic responses [73]. As mentioned previously, leucine has been identified as a particular interesting insulin secretagogue [60,62,66,67] and in an effort to determine the specific role of leucine supplementation an insulinotropic protein mixture was tested together with a single, meal-like, bolus of carbohydrate in longstanding type 2 diabetes patients. Addition of free leucine to the mixture significantly increased circulating insulin concentrations but failed to result in a further improvement in glycemic control [12]. In a series of real life studies, these results have also been confirmed. In these studies the effects of protein modulation (protein only, and protein combined with free leucine) were determined on the prevalence of hyperglycemia in well-controlled type 2 diabetes patients as measured with continuous glucose monitoring. Whereas a protein/leucine mixture was able to lower the prevalence of hyperglycemia by 26% [74], the same absolute amount of protein (without free leucine) did not results in a further improvement of glycemic control [75]. These results extend on previous findings [46,76,77], and imply that nutritional interventions with protein, and leucine in particular can represent an effective strategy to reduce postprandial blood glucose excursions.

However, no long-term studies have focused on the question of whether the insulinotropic potential of amino acids remains after being on a high protein diet or amino acids supplementation. It therefore remains to be seen whether such a nutritional intervention represents a feasible long-term strategy to improve glycemic control. Next to the insulinotropic properties of amino acids and dietary protein there are several other beneficial effects that could result in a better health status for both diabetics, elderly or obese subjects.

In long-term dietary interventions, protein and leucine supplementation would eventually lead to changes in the macronutrient composition of an *ad libitum* diet, while keeping the person in energy balance. The greater protein intake would be accompanied by a reduction in total dietary fat and carbohydrate consumption. This kind of dietary modulation should result in an even further improvement in glycemic control as total carbohydrate intake is lower. In accordance, increasing the protein content of the diet, at the expense of carbohydrate and fat, drastically lowered blood glucose concentrations in a group type 2 diabetes patients over a 5 week intervention period [78,79,80]. Furthermore, it should be noted that diets high in protein have been reported to be more effective when trying to maintain body weight after a period of weight loss when compared to high carbohydrate diets. This benefit has been attributed to the thermogenic and satiating properties of dietary protein [81,82,83,84,85], which in the long run can further optimize glycemic control.

Though there are ample suggestions that amino acid or protein supplementation could represent an effective dietary strategy to improve blood glucose homeostasis in type 2 diabetes, future research should determine if these insulinotropic properties are retained after prolonged increase of dietary protein or BCAA supplementation. 

## 4. BCAA and Muscle Metabolic Health

In addition to the potential for BCAA (and leucine in particular) to benefit metabolic health through hypertrophic or insulinotropic pathways (as described in previous sections), there is emerging evidence that BCAA may also influence skeletal muscle metabolism. Because skeletal muscle is responsible for ~75%–80% of glucose disposal in response to carbohydrate ingestion [87] and is a main contributor to metabolic rate, alterations in skeletal muscle metabolism have profound effects on whole body metabolic health. A loss of skeletal muscle mass, for example, reduces overall glucose disposal capacity, which can result in elevated circulating glucose concentrations, unrelated to the level of insulin sensitivity. Mitochondria are organelles responsible for generating cellular energy through oxidation of substrates and are therefore critical to metabolic regulation. Accordingly, reduced quantity and/or quality of skeletal muscle mitochondria is hypothesized to contribute to insulin resistance in older adults [88] and aging-related diseases, such as type 2 diabetes [89,90]. Mitochondria also play important roles in oxidative stress and apoptosis, which are clearly implicated in the aging process. As such, strategies that can increase, or preserve, muscle mitochondrial function may have therapeutic benefit in aging.

Recently, D’Antona and colleagues [91] fed mice a diet enriched in BCAA and demonstrated that average lifespan was increased. These authors linked the anti-aging effects of BCAA supplementation to increased mitochondrial biogenesis in skeletal muscle and heart, and demonstrated that markers of oxidative stress were reduced. Earlier findings had indicated that dietary leucine supplementation could improve glucose regulation in mice with diet-induced obesity [92]. These beneficial metabolic effects of BCAA supplementation in rodents are supported by some human data, where 60 weeks of AA supplementation (containing relatively high proportion of BCAA) was shown to improve insulin sensitivity and glucose control in a small trial involving elderly patients with type 2 diabetes [93]. Thus, it is possible that BCAA supplementation could have benefits to metabolic health through mechanisms that improve skeletal muscle mitochondria mass and function [94]. 

These potential positive effects of BCAA supplementation for metabolic health and aging must be balanced against any negative outcomes. In this regard, recent human studies have found potential links between elevated plasma BCAA and obesity/type 2 diabetes. Using metabolomics profiling, Newgard and colleagues [95] demonstrated that BCAA were elevated in obese humans, and suggested that BCAA overload may contribute to insulin resistance. In a separate study, circulating BCAA (along with phenylalanine and tyrosine) demonstrated high associations with the development of type 2 diabetes in a group of 2422 individuals who had blood samples taken at baseline and were followed for 12 years [96]. It has been proposed that elevated BCAA concentrations result in an overactivation of mTOR/p70S6 kinase which, in turn, results in an increased IRS-1 phosphorylation on serine residues thereby inhibiting PI3 kinase [94]. This inhibition of PI3K in turn leads to impaired insulin signaling and contributes to insulin resistance [97,98,99]. Future studies are needed to decipher whether these findings are (i) indicative of BCAA contributing to impaired metabolic health, (ii) the result of impaired BCAA catabolism in obesity/diabetes, or (iii) are a possible compensatory mechanism in response to obesity.

## 5. Conclusions

With increasing prevalence of both sarcopenia and type 2 diabetes, there is a great need for novel interventions that effectively combat the loss of skeletal muscle mass and increased insulin resistance that play a pivotal role in both afflictions. Amino acids in general, and the branched-chain amino acids in particular, are likely to represent a nutritional approach that is able to reduce or revert the age-related loss of muscle mass and function, overcome anabolic resistance and improve glycemic control. Leucine consumption increases the activation of the mTOR signaling pathways involved in muscle protein synthesis through insulin-dependent and independent pathways and may therefore overcome the anabolic resistance to nutrition and help to maintain muscle mass in an aging population. Apart from their anabolic properties, amino acids also exhibit strong insulinotropic effects as they can either directly induce insulin secretion or function as a substrate to increase ATP/ADP ratio’s that stimulate insulin exocytosis. The insulinotropic effects of BCAA may exert further influence on positive muscle protein balance by reducing muscle protein breakdown. Furthermore, the same signaling pathways that lead to skeletal muscle protein synthesis also play a role in enhancing β-cell mass and function. BCAA can also influence skeletal muscle metabolism by improving the quantity and quality skeletal muscle mitochondria, and as such, increase, or preserve, muscle mitochondrial function that may have therapeutic benefits in aging. However, more long-term studies are warranted to fully elucidate the true potential of the anabolic and insulinotropic potential of amino acids.
